# Multilevel Analysis of the Association of Dental-Hygienist-Related Factors on Regular Dental Check-Up Behavior

**DOI:** 10.3390/ijerph18062816

**Published:** 2021-03-10

**Authors:** Yuko Inoue, Yoshihiro Shimazaki, Akiko Oshiro, Takashi Zaitsu, Michiko Furuta, Yuichi Ando, Hideo Miyazaki, Masaki Kambara, Kakuhiro Fukai, Jun Aida

**Affiliations:** 1 Department of Oral Health Promotion, Graduate School of Medical and Dental Sciences, Tokyo Medical and Dental University (TMDU), Tokyo 113-8510, Japan; inoue.ohp@tmd.ac.jp (Y.I.); oshiohp@tmd.ac.jp (A.O.); zaitsu.ohp@tmd.ac.jp (T.Z.); 2 Department of Preventive Dentistry and Dental Public Health, School of Dentistry, Aichi Gakuin University, Aichi 464-8650, Japan; shima@dpc.agu.ac.jp; 3 8020 Promotion Foundation, Tokyo 102-0073, Japan; mfuruta@dent.kyushu-u.ac.jp (M.F.); ando.y.aa@niph.go.jp (Y.A.); hideomiy@meirin-c.ac.jp (H.M.); mkamba096@gmail.com (M.K.); fukaik@ka2.so-net.ne.jp (K.F.); 4 Section of Preventive and Public Health Dentistry, Division of Oral Health, Growth and Development, Faculty of Dental Science Kyushu University, Fukuoka 812-8582, Japan; 5 National Institute of Public Health, Saitama 351-0197, Japan; 6 Meirin College, Niigata 950-2086, Japan; 7 Department of Dentistry, Osaka Dental University, Osaka 573-1144, Japan; 8 Fukai Institute of Health Science, Saitama 341-0003, Japan; 9 Division for Regional Community Development, Liaison Center for Innovative Dentistry, Graduate School of Dentistry, Tohoku University, Miyagi 980-8575, Japan

**Keywords:** dental clinic, dental hygienist, dental visit, oral health, regular dental check-ups

## Abstract

Few studies have examined the factors related to dental clinics during dental check-ups. We examined the association between dental-hygienist-related factors and patients’ regular dental check-ups. This nationwide cross-sectional study was based on a survey conducted in Japan in 2014. The analyzed population included 12,139 patients from 1181 dental clinics. We used three-level Poisson regression analysis, considering patient‒clinic‒prefecture, to examine the association of dental-hygienist-related factors with dental check-up behavior. Patients attending treatment and regular check-ups constituted 63.0% and 37.0%, respectively. The adjusted prevalence ratios (PRs) for patients undergoing regular dental check-ups at dental clinics with dedicated dental hygienists’ units, spending ≥20 min in patient education (compared to 0 min), and with three or more dental hygienists (compared to 0 hygienists) available were 1.17 (95% confidence interval [CI]: 1.06–1.30), 1.25 (95%CI: 1.07–1.46), and 2.05 (95%CI: 1.64–2.56), respectively. The median PR indicates that when a patient randomly moves to another dental clinic with more regular dental check-ups, this prevalence increased 1.69 times. These results suggest that dental check-up behavior is determined not only by individual factors but also dental-clinic-level factors. Improving the dental-hygienist-related factors is necessary to encourage people to visit dentists for regular check-ups.

## 1. Introduction

Regular dental check-ups have been reported to have beneficial effects on oral health [1,2] and oral-health-related quality of life (OHRQoL) [3]. They reduce the risk of dental caries [4] and periodontal disease [5]. Dental caries and periodontal disease are major causes of tooth loss [6,7], and regular dental check-ups contribute to the maintenance of natural teeth. In aging societies, maintaining natural teeth in older age is considered to be important for extending healthy life expectancy [8].

Previous studies have examined factors related to behavior regarding regular dental check-ups. It has been reported that men [9], people with lower socioeconomic status [10,11,12,13], and those with dental anxiety [14] tend to avoid regular dental check-ups. However, very few studies have examined factors related to dental clinics, rather than patients.

Dental hygienists are experts in preventive oral care and play an important role in regular dental check-ups by providing preventive dental services and oral health education [15,16,17,18]. Therefore, dental hygienists’ workforce-related factors, such as the number of dental hygienists at dental clinics, the presence of dental hygienists’ dedicated dental units, and duration of dental hygienists’ patient education, may affect patients’ dental visit behavior. Surveys of dental clinics have suggested that dental clinics with more dental hygienists were positively associated with regular dental check-ups [19]. However, very few studies have examined the association between the number of dental hygienists and patients’ regular dental check-ups. Additionally, to the best of our knowledge, there have been no reports on the association of dental-hygienist-related factors, such as the availability of a dental unit for dental hygienists in a clinic, or the average time spent in oral health education of a patient in a clinic, and whether patients regularly attend dental check-ups. A dental hygienist unit makes it possible for a dental clinic to provide separate appointments with a dental hygienist, rather than with a dentist, to provide intensive cleaning and oral health education.

Therefore, the present study aimed to examine the association of dental-hygienist-related factors with patients’ dental check-up behavior. We hypothesized that patients with access to a dental clinic with dental hygienists, dental units dedicated to dental hygienists, and where longer time was spent on oral health education would tend to attend the dental clinic regularly.

## 2. Methods

### 2.1. Data Source

For this cross-sectional study, we used data from the “Study on the Health Promotion Effects of Dental Care”, conducted by the 8020 Promotion Foundation in 2014 [20,21,22,23,24]. This study was composed of two surveys—a survey of dental clinics and a survey of their patients—and aimed to measure dental care services in dental clinics, evaluate the oral health of patients, and assess the factors that affect their access to dental care. The survey questionnaire is shown in Supplementary Figure S1. In 2014, among Japan’s 47 prefectures, dental associations in 46 prefectures agreed to participate in the study. Among the 1354 selected dental clinics, 1216 clinics replied to the surveys (response rate = 89.8%). These dental clinics responded to a questionnaire survey in the clinic. Patients aged 20 years and older who visited these dental clinics for a week in October 2014 were asked to participate in the survey: 12,604 first-time and returning patients of these clinics completed the questionnaire. After excluding respondents who did not state the reason for their dental visits (*N* = 465), we used data from 12,139 patients at 1181 dental clinics. 

### 2.2. Dependent Variables: Reason for the Dental Visit

The reasons for dental visits were obtained from a survey of dental patients. The question was, “What is the reason for your visit to the dental clinic?”, with the responses, “for treatment,” “for regular check-ups,” and “for treatment and regular check-ups.” These responses were dichotomized as follows: (0) treatment with or without regular check-ups, and (1) regular check-ups only. In the sensitivity analysis, we dichotomized the responses regarding dental check-ups as follows: (0) treatment and (1) regular check-ups, with or without treatment. Then, we conducted the same analyses as the main analyses.

### 2.3. Independent Variables: Dental-Hygienist-Related Factors

Information on the dental-hygienist-related factors was obtained from a survey of dental clinics. The survey inquired about the number of dental hygienists who worked full-time and part-time at each dental clinic. We calculated the “number of dental hygienists in the clinic” as the sum of the number of full-time hygienists and the number of part-time hygienists, weighted by their work hours. We also asked about the number of dental units dedicated to dental hygienists at the clinic. This number was classified into 2 categories (0 units, ≥1 unit). The time spent on oral health education for each patient was also assessed. The responses were divided into 4 categories (0, 1–9, 10–19, and ≥20 min).

### 2.4. Covariates

We used several covariates based on previous studies: age (20–29, 30–39, 40–49, 50–59, 60–69, 70–79, 80–89, and ≥90 years), sex, subjective economic status (high, upper-middle, middle, low-middle, low), and years of education (≤9, 10–12, 14–15, ≥16). The subjective economic status was queried using the following question: “Please choose the number that best describes your family’s current financial situation, assuming that the average family is “middle”. The choices were “high”, “upper-middle”, “middle”, “low-middle”, and “low” (please see Supplementary Figure S1). The responses regarding subjective economic status were aggregated because there were few responses for extreme categories; the “high” and “upper-middle” categories were combined into a “high” category, and the “low-middle” and “low” categories were combined into a “low” category. The number of dentists, which was determined in the same way as the number of dental hygienists, was also used as a covariate.

### 2.5. Ethical Considerations

Informed consent was obtained from all study participants. The Ethics Committee of the Japan Dental Association approved the study design, data collection method, and procedures for obtaining informed consent (approval number 002).

### 2.6. Statistical Analysis

We applied Poisson regression to estimate the prevalence ratio (PR) as the prevalence of dental check-ups was higher, and the odds ratio estimated by logistic regression overestimates the association [25]. The data derived from each prefecture and multiple dental clinics were structured by 3 nested strata, namely prefectures, dental clinics, and patients; therefore, a 3-level multilevel Poisson regression analysis was used to examine the association between dental-hygienist-related factors and patients’ dental visit behavior regarding check-ups by using prevalence ratios (PRs) and 95% confidence intervals (CIs). Attendance at a dental check-up was used as the dependent variable. For the dental-hygienist-related factors, 3 variables, i.e., the number of dental hygienists in a clinic, the presence of dental units for the dental hygienist, and the average time spent on oral health education for patients, were included as the independent variables. We adjusted for the patients’ age, sex, subjective economic status, year of education, and the number of full-time dentist-equivalents as covariates. Variables on the 3 dental-hygienist-related factors were correlated, and to test which dental-hygienist-related factor accounted for regular dental check-ups, each dental-hygienist-related factor was separately added to Models 1 to 3, respectively. In Model 4, all 3 dental-hygienist-related factors were added to all covariates. From the multilevel analysis, we also calculated the variance partition coefficient (VPC) and median prevalence ratio to represent the percentage variance of regular dental check-ups explained by dental clinics and prefecture [26]. For missing values, we used multiple imputations (MIs) [27]. In the present study, we replaced each missing value with a set of substituted plausible values by creating 10 filled-in complete datasets using MI with a chained equation method. In the imputation process, the following variables were used to create 10 complete datasets: age, sex, subjective economic status, dental unit for dental hygienists, amount of time spent on oral health education, the number of full-time dentist-equivalents, and the number of full-time dental-hygienist-equivalents. All data analyses were conducted using STATA^®^ 15.1 (Stata Corporation, College Station, TX, USA). *p* < 0.05 indicated statistical significance.

## 3. Results

Table 1 shows the descriptive characteristics of the patients, according to the reason for the dental visit, after applying MIs. Among 12,139 patients, 63.0% (7651/12,139) reported treatment-related dental visits, and 37.0% (4488/12,139) reported dental visits for regular check-ups. Patients attending dental clinics with one or more dental units dedicated to dental hygienists, where a longer time was spent in oral health education, or with:

A larger number of dental hygienists or dentists tended to undergo regular dental check-ups. In terms of patient characteristics, females and those with high economic status and high education level tended to undergo dental check-ups regularly. Patients aged 80 years or older reported a lower prevalence of regular dental check-ups.

Table 2 shows the PRs for regular dental visits, rather than treatment-related dental visits, estimated from the three-level multilevel Poisson regression analysis after applying MI. In Model 1, even after adjusting for the covariates, patients attending dental clinics equipped with dedicated dental hygienists’ units had a significantly higher PR of undergoing dental check-ups than those attending clinics without any units (PR 1.24, 95%CI: 1.11–1.37). According to Model 2, patients attending clinics that spent more time on oral health education had a higher PR for undergoing dental check-ups than those attending clinics that did not conduct oral health education. The PR was higher in the dental clinics with a longer education time. In Model 3, patients attending clinics with a larger number of dental hygienists showed higher PR for regular dental check-ups. To consider the association of each dental hygienist factor, all variables were included in Model 4. All factors still showed significant associations, although the PR was decreased compared to previous models. In this model, the number of dentists did not show any significant associations. Sensitivity analysis using different categorizations of the dental check-ups variables also showed similar results (Supplementary Tables S1 and S2).

Throughout the multilevel models, the difference in regular dental check-ups was larger between dental clinics than between prefectures. The VPC from the null model showed that 8.4% of the variation in regular dental check-ups occurred between dental clinics, and 0.6% of the variation occurred between prefectures. The median PR for the null model indicates that, when a patient randomly moves to another dental clinic or prefecture with more regular dental check-ups, the median of the PR for the patient to attend dental clinics regularly is 1.69 (95%CI: 1.60–1.79) for dental clinics and 1.14 (95%CI: 1.10–1.21) for prefectures. The “dental units dedicated to dental hygienists” explained the largest VPC of dental clinics (VPC of model 1 = 7.3%) compared to “the time spent on oral health education” (VPC of model 2 = 7.1%) and “number of dental hygienists” (VPC of model 3 = 6.0%). This suggested that the presence of dental units dedicated to dental hygienists was the factor that contributed most to the difference in regular dental check-ups between clinics. Even after adjusting for all variables, the VPC in Model 4 was not substantially lower than that in Model 1.

## 4. Discussion

This study examined the association of dental-hygienist-related factors with patients’ regular dental check-up behavior, using a nationwide survey conducted in Japan. To the best of our knowledge, no previous study has examined the impact of various dental-hygienist-related factors on patients’ regular dental check-up behavior. Our results suggested that dental clinics employing a larger number of dental hygienists, equipped with dental units dedicated to dental hygienists, and spending a longer period of time on oral health education tended to have more patients attending regular dental check-ups than clinics that did not. In addition, three-level multilevel logistic regression analysis revealed that a large part of the difference in check-up behavior was related to differences between clinics rather than between prefectures. Additionally, the presence of dental units dedicated to dental hygienists was the largest contributing factor to the differences between clinics.

Previous studies have also shown a positive association between clinics with dental hygienists available and patients regularly attending dental check-ups [28]. The provision of proactive prevention to patients tended to depend on the employment of dental hygienists in practice, and patients attending dental clinics where dental hygienists were available had significantly more active prevention than those in clinics without dental hygienists [28]. In addition, the present study also showed that, even after adjusting for the number of dental hygienists at a clinic, patients tended to attend regular dental check-ups in clinics equipped with dental units dedicated to dental hygienists. This is consistent with the results of a previous study in Germany, which reported that the presence of a specialized preventive unit in a clinic was significantly associated with the provision of preventive care [29]. It is considered that dental clinics with dental units dedicated to dental hygienists are able to use more time for education, which increases the patients’ check-up behavior.

Degrees of the observed association of dental-hygienist-related factors with dental check-ups shown by PRs were comparative or higher than subjective economic status or educational attainment. Many previous studies reported social inequalities in access to dental care [10,11,12,13]. However, the present result showed a stronger association of dental-hygienist-related factors. Therefore, these factors should be considered as important social determinants of access to dental care.

The present results imply that addressing dental-hygienist-related factors at dental clinics could promote regular dental visits. The dental hygienist’s role in dental clinics is also beneficial for the oral health of patients. A previous study using the same dataset as the present study suggested that a larger number of dental hygienists and a longer period of time spent on oral health education significantly reduced the risk of tooth loss [24]. In Japan, the public dental insurance system mainly focuses on dental treatment, rather than on prevention [30]. In addition, the access of patients to preventive care depended on their financial situation [31]. Therefore, public insurance should cover more preventive care. However, there is still room for improvement in dental clinics in Japan. According to a nationwide survey by the Japan Dental Hygienists Association, the number of private dental clinics with dedicated units for dental hygienists was 29.5% in 2010 but was 36.0% in 2020 [32]. This increment could extend the time available for dental education and improve the dental health behavior of patients. Policies that encourage preventive attitudes in dental clinics should be promoted to increase the number of such clinics in the future.

This study had several strengths and limitations. A strength of this study is that we used both patient- and dental-clinic-level data. This shed light on the influence of the clinic-related factors on patients’ behavior. In addition, because this survey was a nationwide survey, we could determine between-prefecture differences and between-clinic differences in regular dental check-ups. However, several limitations of this study need to be addressed. First, the clinics considered in this study were selected from clinics that belonged to dental associations, rather than randomly selected clinics. This reduces the generalizability of the results. Second, the cross-sectional design of this study makes it difficult to infer causal relationships. It is possible that an increase in the number of patients attending preventive visits will increase the number of dental hygiene units or extend the instructional time for dental hygiene instruction. Third, we did not examine dentists’ specialties. Specialties can affect treatment patterns. However, because there would be nonsystematic bias for sampling the specialty of the clinics, non-differential misclassification could attenuate the observed association. Finally, most dental treatments in Japan are covered by public medical insurance; however, it is not clear whether the regular dental check-ups were covered by insurance or were self-financed. It is necessary to investigate this subject in more detail in the future.

## 5. Conclusions

In conclusion, we found that dental clinics that employed a larger number of dental hygienists, had dedicated dental units available for dental hygienists, and spent a longer period of time on oral health education tended to have more patients attending regular dental check-ups. These dental-hygienist-related factors largely explained the differences in the check-up behavior of patients. PRs of several dental-hygienist-related factors and MPR of dental clinics were larger than the PRs of economic status and educational background. We propose that increasing the number of hygienists and improving the working environment and the optimal use of these professionals is necessary to encourage people to visit dentists for regular check-ups.

## Figures and Tables

**Table 1:** Characteristics of participants according to the reason for dental visit.

		Treatment *N* = 7651 (63.0%)		Dental Check-Ups *N* = 4488 (37.0%)		*p*-Value
		*N*	%	*N*	%	*p*
Dental units dedicated to dental hygienists						
	None	5921	65.6	3102	34.4	*p* < 0.01
	≥1 dental unit	1730	55.5	1386	44.5	
Time spent on oral health education						
	0 min	1510	71.9	590	28.1	*p* < 0.01
	1–9 min	2531	63.1	1483	37.0	
	10–19 min	2394	60.7	1553	39.3	
	≥20 min	1217	58.5	862	41.5	
Number of dental hygienists (full-time equivalent)						
	0	1072	78.2	298	21.8	*p* < 0.01
	<0–0.9	1833	72.5	697	27.6	
	1–1.9	1734	64.2	967	35.8	
	2–2.9	1373	57.1	1031	42.9	
	≥3	1639	52.3	1,495	47.7	
Number of dentists (full-time equivalent)						
	1	4728	66.0	2441	34.1	*p* < 0.01
	<1–1.9	2003	59.2	1383	40.8	
	≥2	920	58.1	664	41.9	
Sex						
	Male	2950	68.1	1381	31.9	*p* < 0.01
	Female	4701	60.2	3107	39.8	
Age (years)						
	20–29	582	59.6	394	40.4	*p* <0.01
	30‒39	903	60.3	595	39.7	
	40‒49	1136	60.3	747	39.7	
	50‒59	1341	64.5	737	35.5	
	60–69	1826	63.1	1066	36.9	
	70–79	1456	64.9	786	35.1	
	80–89	386	70.7	160	29.3	
	≥90	21	87.5	3	12.5	
Subjective economic status						
	Low	1642	68.3	763	31.7	*p* < 0.01
	Middle	5049	62.1	3,086	37.9	
	High	960	60.1	639	39.9	
Years of education						
	≤9	724	72.3	277	27.7	*p* < 0.01
	10–12	3344	64.9	1810	35.1	
	14–15	2122	59.8	1430	40.3	
	≥16	1460	60.1	971	39.9	

**Table 2:** Prevalence ratio (PR), median prevalence ratio, and variance partition coefficient for regular dental check-ups.

		Crude				Model 1				Model 2				Model 3				Model 4			
		PR	95%CI			PR	95%CI			PR	95%CI			PR	95%CI			PR	95%CI		
Fixed effects																					
Dental units dedicated to dental hygienists																					
	None	1.00				1.00												1.00			
	≥1 dental unit	1.29	1.17	-	1.43	1.24	1.11	-	1.37									1.17	1.06	-	1.30
The time spent on oral health education																					
	0 min	1.00								1.00								1.00			
	1–9 min	1.30	1.12	-	1.50					1.24	1.08	-	1.42					1.18	1.03	-	1.35
	10–19 min	1.41	1.23	-	1.62					1.31	1.16	-	1.49					1.19	1.05	-	1.36
	≥ 20 min	1.50	1.28	-	1.75					1.40	1.20	-	1.63					1.25	1.07	-	1.46
Number of dental hygienists (full-time equivalent)																					
	0	1.00												1.00				1.00			
	<0–0.9	1.28	1.07	-	1.53									1.28	1.07	-	1.55	1.23	1.01	-	1.50
	1–1.9	1.66	1.35	-	2.04									1.61	1.31	-	1.98	1.51	1.23	-	1.86
	2–2.9	1.96	1.60	-	2.41									1.92	1.57	-	2.35	1.82	1.48	-	2.24
	≥3	2.28	1.85	-	2.83									2.18	1.75	-	2.72	2.05	1.64	-	2.56
Number of dentists (full-time equivalent)																					
	1	1.00				1.00				1.00				1.00				1.00			
	<1–1.9	1.17	1.04	-	1.30	1.14	1.03	-	1.27	1.12	1.01	-	1.25	1.04	0.94	-	1.16	1.03	0.93	-	1.14
	≥2	1.23	1.08	-	1.40	1.15	1.01	-	1.31	1.14	0.99	-	1.31	0.95	0.83	-	1.10	0.91	0.79	-	1.04
Sex																					
	Male	1.00				1.00				1.00				1.00				1.00			
	Female	1.25	1.17	-	1.33	1.20	1.13	-	1.26	1.19	1.13	-	1.26	1.20	1.14	-	1.26	1.20	1.13	-	1.26
Age (years)																					
	20–29	1.00				1.00				1.00				1.00				1.00			
	30–39	0.98	0.68	-	0.88	0.97	0.88	-	1.06	0.97	0.88	-	1.07	0.96	0.87	-	1.06	0.96	0.88	-	1.06
	40–49	0.98	0.57	-	0.90	0.99	0.91	-	1.07	0.99	0.91	-	1.08	0.99	0.91	-	1.07	0.99	0.91	-	1.07
	50–59	0.88	0.02	-	0.78	0.89	0.80	-	0.99	0.89	0.80	-	0.99	0.90	0.81	-	1.00	0.90	0.80	-	1.00
	60–69	0.90	0.08	-	0.80	0.94	0.85	-	1.04	0.94	0.85	-	1.04	0.95	0.86	-	1.05	0.95	0.86	-	1.05
	70–79	0.86	0.02	-	0.76	0.93	0.83	-	1.03	0.93	0.83	-	1.03	0.94	0.84	-	1.05	0.94	0.85	-	1.05
	80–89	0.72	0.00	-	0.60	0.83	0.71	-	0.96	0.83	0.72	-	0.96	0.85	0.73	-	0.99	0.85	0.73	-	0.99
	≥90	0.31	0.04	-	0.10	0.46	0.16	-	1.30	0.46	0.16	-	1.30	0.46	0.16	-	1.30	0.47	0.17	-	1.31
Subjective economic status																					
	Low	1.00				1.00				1.00				1.00				1.00			
	Middle	1.19	1.10	-	1.28	1.13	1.06	-	1.21	1.13	1.05	-	1.21	1.12	1.04	-	1.20	1.12	1.04	-	1.20
	High	1.23	1.11	-	1.38	1.13	1.04	-	1.24	1.13	1.03	-	1.24	1.13	1.03	-	1.23	1.12	1.03	-	1.23
Years of education																					
	≤9	1.00				1.00				1.00				1.00				1.00			
	10–12	1.26	1.13	-	1.41	1.13	1.01	-	1.25	1.13	1.01	-	1.25	1.13	1.02	-	1.26	1.13	1.02	-	1.26
	14–15	1.43	1.28	-	1.60	1.20	1.08	-	1.34	1.20	1.08	-	1.34	1.20	1.08	-	1.34	1.20	1.08	-	1.34
	≥16	1.42	1.27	-	1.59	1.25	1.13	-	1.39	1.25	1.13	-	1.39	1.26	1.13		1.39	1.26	1.13	-	1.40
Random effects (SE)																					
Prefecture		0.02 *	(0.01)			0.01	(0.01)			0.02	(0.01)			0.02	(0.01)			0.02	(0.01)		
Dental clinic		0.30 *	(0.03)			0.26	(0.03)			0.25	(0.03)			0.21	(0.02)			0.19	(0.02)		
Median prevalence ratio (MPR)		MPR	95%CI			MPR	95%CI			MPR	95%CI			MPR	95%CI			MPR	95%CI		
Prefecture		1.14 *	1.10	-	1.21	1.10	1.10	-	1.21	1.14	1.10	-	1.21	1.14	1.10	-	1.21	1.14	1.10	-	1.18
Dental clinic		1.69 *	1.60	-	1.79	1.63	1.55	-	1.72	1.61	1.55	-	1.70	1.55	1.55	-	1.72	1.52	1.45	-	1.60
Variance partition coefficient (VPC)																					
Prefecture		0.6% *				0.3%				0.6%				0.6%				0.6%			
Dental clinic		8.4% *				7.3%				7.1%				6.0%				5.5%			

* Random effects, MPR, and VPC for the null model; the model includes only a constant.

## Data Availability

The datasets used and analyzed during the current study are available from the corresponding author on reasonable request.

## Supplementary Materials

The following are available online at https://www.mdpi.com/1660-4601/18/6/2816/s1, Table S1: Characteristics of participants by the reason for dental visit, Table S2: Prevalence ratio (PR), Median prevalence ratio, and variance partition coefficient for regular dental check-ups. Figure S1: The survey questionnaire was translated into English.
