# Supplementary material for: Multilevel Analysis of the Association of Dental-Hygienist-Related Factors on Regular Dental Check-Up Behavior
Source: Int J Environ Res Public Health. 2021 Mar 10;18(6):2816. doi: 10.3390/ijerph18062816 (PMC7998093; doi:10.3390/ijerph18062816)
Supplement: Supplementary file 1 [file ijerph-18-02816-s001.zip › ijerph-1118612_supplementfigure.pdf]

Supplemental Figure S1 .The survey questionnaire was translated into English.

Dental Health Examination Form

DATE : year month day

Patient name ________________________________

Dentition status

	8	7	6	5	4	3	2	1	1	2	3	4	5	6	7	8	
R																	L
	8	7	6	5	4	3	2	1	1	2	3	4	5	6	7	8	

The number of healthy teeth (/)	The number of caries(C)	The number of filling teeth(○)	The number of teeth (sum)	The number of missing and need to treatment teeth(△)	The number of fixed dental prosthesis(◎)

【CPI improved】

	18	17	16	15	14	13	12	11	21	22	23	24	25	26	27	28
BOP																
PPD																
BOP																
PPD																
	48	47	46	45	44	43	42	41	31	32	33	34	35	36	37	38

Description of CPI Improved Code
Bleeding column:
None = 0, Yes = 1, Not measurable = 9, Loss of tooth = X
Periodontal pocket (PD) column
None = 0, PD 4-5 mm = 1, PD 6 mm or more = 2, Not measurable = 9, Tooth loss = X

	17/16	11	26/27
Attachment loss			
	47/46	31	36/37

Attachment loss
In the depth of the pocket from the cement enamel border
0 to 3 mm = 0 ・ 4 to 5 mm = 1 ・ 6 to 8 mm = 2
9 to 11mm = 3 ・ 12mm or more = 4
Unmeasurable = 9 ・ Tooth loss = X

Questionnaire for dental Clinics

DATE :　　　　year　　　　month　　　　day

The name of dental clinics ______________________

Q 1 . Please fill the address your dental clinic.

Zip code　　—

____

Q2: Please write the gender of the director of your clinic.

1. Male　　　2. Female

Q3. Please write the age of the director of your clinic.

______________ Years old

Q4. Please circle all that apply to your clinic's advocated department.

1. Dentistry　2. Pedodontics　3. Orthodontics　4. Dental surgery

Q5. Please write the number of examination tables (units) and the number of dental units dedicated for dental hygienists in your clinic.

Number of units　　　______ units

(Of these, the number of dental units dedicated to dental hygienists) ______ units

Q6. Please write the number of patients on the day you filled out this questionnaire.

①Number of first-time patients　　______________ people

②Number of follow-up patients　　______________ people

Q 7 .Please write the number of full-time and part-time staff in your clinic.
(If you have no staff, please write "0".)

	Full-time	Part-time	
	The number of staff	The number of staff	Number of full-time equivalent
Dentist(including yourself)	people	people	people
Dental hygienist	people	people	people
Dental technician	people	people	people
Dental assistant	people	people	people
Others	people	people	people

※The number of part-time workers should be calculated on a full-time basis (see the table below).

【Example】　In the case of a 5-day work week at your hospital

If you work all day 5 days a week: 5/5 x 1 = 1 person

If you work one full day per week: 1/5 x 1 = 0.2

Half-day work 5 days a week: 5/5 x 0.5 = 0.5

Q8: In the past month, did you provide any of the following preventive dental care services? (Please circle one for each item)

		1<br>Yes	2<br>No
Fluoride applications		1	2
Fluoride mouthwash		1	2
Sealant		1	2
Prevention and control		1	2
	Of which, continuous control for more than 1 year	1	2

Q9. Have you had a regular checkup in the past month? (Circle one)

1.Yes → Go to Q9-1 2.No → Go to Q10

【If you answered "1.Yes" to Q9, please answer.】

Q9-1. How often do you provide treatment and instructions to adults during regular dental checkups for each item? (Circle one for each item)

		1<br>Always every time	2<br>Often	3<br>Not very seldom.	4<br>Not at all
Oral examination	Tooth (caries, etc.)	1	2	3	4
	Periodontal	1	2	3	4
Tooth scaling.	Supragingival	1	2	3	4
	Subgingival	1	2	3	4
Tooth cleaning		1	2	3	4
Fluoride application		1	2	3	4
Fluoride mouthwash instruction		1	2	3	4
Oral health instruction (Tooth brushing instruction, etc.)		1	2	3	4
Dietary instruction		1	2	3	4
Smoking cessation instructions		1	2	3	4
Other ( )		1	2	3	4

Q10: Have you provided dental health instructions in the past month? (Circle one)

1. Yes, I did → Go to Q10-1 2. No, I did not → End of question

Q10-1. How much time do you spend on average for each oral health instruction for each patient? Please give us a rough estimate. (Circle one)

1. Less than 5 minutes 2. 5 to 9 minutes 3. 10 to 19 minutes
4. 20 to 29 minutes 5. 30 minutes or more 6. Don't know

Thank you for your cooperation.

Patient Questionnaire

DATE :   year   month   day

Patient name ____ Sex(Male・Female)

Age   years old   Date of birth :   year   month   da

Address

____

Q1-1. How would you describe the state of your teeth and gums?
1. Good   2. Fairly good   3. Average   4. Not so good   5. Poor

Q1-2 Do you have any concerns about the condition of your teeth and mouth?
1. Chewing   2. Appearance   3. Speech   4. Halitosis
5. Pain (Please choose one→ Teeth, Gums, Tongue, Others.)   6. Denture is not good
7. Difficulty swallowing   8. Dry mouth   9. Interruption of dental treatment
10. Jaw joint   11. Teeth alignment   12. Others   13. No particular problem

Q2-1 How would you describe the state of your overall health?
1. Good   2. Fairly good   3. Average   4. Not so good   5. Poor

Q2-2: Do you have any of the following symptoms at present?
Please circle all that apply.
1. Shoulder pain   2. Back pain   3. Elbow or hand pain   4. Foot pain
5. Neuralgia   6. Tinnitus   7. Hearing loss   8. Blurred vision
9. Insomnia   10. Other (symptoms:)   11. Nothing in particular

Q2-3 I would like to ask about your health.

①Do you currently receive treatment for any of the following diseases?
(Circle all that apply)

1. Diabetes   2. Stroke   3. Heart disease   4. Cancer   5. Pneumonia
6. Hypertension   7. Hyperlipidemia   8. Obesity   9. Dementia
10. Chronic occlusive pulmonary disease   11.Other (   )
12. No particular disease

②Have you ever had a disease in the past for which you are not currently receiving treatment? (Circle all that apply)

1. Diabetes   2. Stroke   3. Heart disease   4. Cancer   5. Pneumonia
6. Hypertension   7. Hyperlipidemia   8. Obesity   9. Dementia
10. Chronic occlusive pulmonary disease   11.Other (   )
12. No particular disease

③Have you ever been diagnosed with any diseases that are not treated now, but were pointed out in your medical checkup?
1. Diabetes 2. Stroke 3. Heart disease 4. Cancer 5. Pneumonia
6. Hypertension 7. Hyperlipidemia 8. Obesity 9. Dementia
10. Chronic occlusive pulmonary disease 11.Other ( )
12. No particular disease
13. I don't know, because I haven't had a medical checkup.

Q2-4 Have you been certified for nursing care? (If you are in the process of applying, please indicate your current certification status.)
1. No 2. Requiring help 1 3. Requiring help 2
4. Long-term care level 1 5. Long-term care level 2 6. Long-term care level 3
7. Long-term care level 4 8. Long-term care level 5

Q2-5 Because of the state of your teeth or mouth, how often have you any problems with your work during the past year?
1. No 2. Occasionally 3.Sometimes 4. Often.

Q2-6 Because of the state of your teeth or mouth, how often have you interrupt your sleep during the past year?

1. No 2. Occasionally 3.Sometimes 4. Often.

Q3-1. How many teeth do you have? (Crown (gold or silver teeth), false teeth, and teeth with only the roots remaining are included in the number of teeth. If you have all your teeth, you have 28-32 teeth (there are up to 4 individual differences in the number of wisdom teeth).
Number of teeth : ( ) teeth

Q3-2. Please tell us about your use of dentures.
(1) Do you have any removable partial dentures?
1. Upper jaw 2.Lower jaw 3.Not used
(2) Do you have any removable complete dentures?
1. Upper jaw 2.Lower jaw 3.Not used

Q4. Can you chew your right and left back teeth firmly with your own teeth or dentures?
1. Both left and right teeth 2. One side 3. Neither of side can chew.

Q5. How often do your teeth bleed when you brush them?
1. Always 2. Sometimes 3. No

Q6. How often do you feel your gums swollen?
1. Always 2. Sometimes 3. No

Q7. How often do you feel your teeth sensitive to cold or hot foods?
1. Always 2. Sometimes 3. No

Q8. Do you have a dental office that you visit regularly?
1. Yes 2. No

Q9. Do you have difficulty going to the dentist because of your work?
1. Yes 2. No

Q10. Are your family members or other people around you interested in oral health?
1. Yes 2. Neither 3. No

Q11. Do you have confidence in your teeth or have you ever been praised by others?
1. Yes 2. Neither 3. No

Q12. How often do you brush your teeth at work or when you go out?
1. Every time 2.Sometimes 3.Never

Q13. How often do you have snacks (sweet foods and drinks)?
1. Every time 2.Sometimes 3.Never

Q14. Please tell us about your daily habits.
①Tobacco
1. Current smoking 2. Ex-smoking 3.Never
②Alcohol
1. Every day 2. At least once a week 3. Less than once a week
4. Quit drinking 5. Never
③Exercise habits
1. Every day 2. Several times a week
3. Several times a month 4. Less than once a month
④Vegetable intake
1. At least twice a day 2. At least once a day
3. Several times a week 4. Less than once a week

Q15-1. How often do you clean your teeth?
1. 3 times or more a day 2. Twice a day 3.Once a day
4. Less than a few times a week 5. Never

Q15-2. How often do you clean your teeth at night before going to bed?
1. Every day 2.Sometimes 3.Never

Q15-3. Do you use toothpaste when you brush your teeth?
1. Fluoride toothpaste 2. Non-fluoride toothpaste.
3. I don't know if it contains fluoride, but I use toothpaste. 4Never

Q16. How often do you use an interdental brush or floss (thread toothpick)?
1. Every day 2.Sometimes 3.Never

Q17. How often do you chew your food slowly and well?
1. Every day 2.Sometimes 3.Never

Q18. What is the reason for your visit to the dental clinic?
1. For treatment 2.For regular checkup 3.Treatment and regular checkup

Q19. Have you visited a dentist in the past?
1. Yes 2.No

[If you answered yes to Q19, please answer the following questions.]
Q19-1. When was the last time you went to the dentist?
1. Within 3 months 2. Within 6 months 3. Within 1 year 4. Within 2 years
5. Within 3 years 6. Within 5 years 7. More than 5 years ago
Q19-2. What is the reason for your visit to the dental clinic for last time?
1. For treatment 2.For regular checkup 3.Treatment and regular checkup
[If you answered 2 or 3 to Q19-2, please answer the following questions.]
Q19-3. How often do you have regular checkups?
1. Once in 6 months 2. Once within 1 year
3. Once every two to three years 4. Other than that

Q20: Have you ever received tooth brushing instruction at a dentist's office?
1. Yes 2.No

Q21: Do you have anyone who can help you when you are in trouble? Please select all that apply.
1. Family 2. Friends 3. Colleagues at work 4. Neighbors
5. Members of a hobby or volunteer group 6. Others 7. No one

Q22-1: Do you work now?
1. I am working 2. I am not working

[If you answered "1. I am working" to Q22-1, please answer the following questions.]
Q22-2: What is your occupation?
1. Manager/ Director 2. Full-time employee (manager)
3. Full-time employees (general employees) 4. Part-time employees
5. Dispatched workers, contract workers, temporary employees
6. Self-employed, freelancer 7. Others ( )
Q22-3: Which of the following types of work do you do?
1. Professional and technical occupations 2. Managerial occupations
3. Clerical and sales occupations 4. Sales and service occupations
5. Security occupations 6. Labor and factory work occupations
7. Agriculture, forestry, and fishing 8.Others ( )

Q23. Please circle the answer that best describes your family's current economic status, assuming that the average family is in the "middle" category.
Please be sure to state your own subjective opinion.
1. Upper 2. Upper middle 3. Middle 4. Lower middle 5. Lower

Q24: How many years have you been attending school? (Please count from the time you entered elementary school.)
1. 9 years or less 2. 10 to 12 years 3. 13 to 15 years 4. 16 years or more

Q25: Please fill in your height and weight.
Height________________cm, Weight______________kg

Thank you very much for your cooperation.
