# Supplementary material for: Multilevel Analysis of the Association of Dental-Hygienist-Related Factors on Regular Dental Check-Up Behavior
Source: Int J Environ Res Public Health. 2021 Mar 10;18(6):2816. doi: 10.3390/ijerph18062816 (PMC7998093; doi:10.3390/ijerph18062816)
Supplement: Supplementary file 1 [file ijerph-18-02816-s001.zip › ijerph-1118612_supplementtable0309.pdf]

Supplemental Table S1. Characteristics of participants by the reason for dental visit

	Treatment N=5,734(47.2%)		Dental check-ups N=6,405(52.8%)		p-value
	N	%	N	%	P
Dental units dedicated to dental hygienists					
None	4,480	49.7	4,542	50.3	p<0.01
≧1　dental unit	1,254	40.2	1,863	59.8	
The time spent on oral health educations					
0 minute	1,181	56.2	919	43.8	p<0.01
1-9 minutes	1,906	47.5	2,108	52.5	
10-19 minutes	1,767	44.8	2,178	55.2	
20 minutes or more	880	42.3	1,200	57.7	
Number of dental hygienists (full time equivalent)					
0 person	881	64.3	489	35.7	p<0.01
<0 - 0.9	1,450	57.3	1,079	42.7	
1 - 1.9	1,307	48.3	1,397	51.7	
2 - 2.9	1,006	41.9	1,397	58.1	
3 people or more	1,091	34.8	2,043	65.2	
Number of dentists (full time equivalent)					
1 person	3,645	50.8	3,525	49.2	p<0.01
<1-1.9	1,450	42.8	1,935	57.2	
2 people or more	639	40.3	945	59.7	
Sex					
Male	2,288	52.8	2,043	47.2	p<0.01
Female	3,446	44.1	4,362	55.9	
Age(years)					
20-29	453	46.4	523	53.6	p<0.01
30-39	689	46.0	809	54.0	
40-49	895	47.5	988	52.5	
50-59	1,016	48.9	1,062	51.1	
60-69	1,317	45.5	1,575	54.5	
70-79	1,069	47.7	1,173	52.3	
80-89	279	51.1	267	48.9	
90+	16	66.7	8	33.3	
Subjective economic status					
Low	1,277	53.1	1,129	46.9	p<0.01
Middle	3,796	46.7	4,339	53.3	
High	662	41.4	937	58.6	
Years of education					
≦9	579	57.7	424	42.3	p<0.01
10-12	2,551	49.5	2,605	50.5	
14-15	1,553	43.7	1,997	56.3	
≧16	1,052	43.3	1,378	56.7	

Supplemental Table S2. Prevalence ratio(PR), Median prevalence ratio, and variance partition coefficient for regular dental check-ups.*

	Crude		Model 1		Model 2		Model 3		Model 4	
	PR	95%CI	PR	95%CI	PR	95%CI	PR	95%CI	PR	95%CI
Fixed-effects										
Dental units dedicated to dental hygienists										
None	1.00		1.00						1.00	
1 dental unit or more	1.19	1.11 - 1.28	1.15	1.08 - 1.23					1.11	1.04 - 1.18
The time spent on oral health educations										
0 minute	1.00				1.00				1.00	
1-9 minutes	1.19	1.07 - 1.32			1.14	1.03 - 1.26			1.10	0.99 - 1.22
10-19 minutes	1.26	1.14 - 1.40			1.19	1.08 - 1.32			1.11	1.00 - 1.23
20 minutes or more	1.33	1.20 - 1.49			1.26	1.14 - 1.39			1.15	1.04 - 1.28
umber of dental hygienists (full time equivale										
0 person	1.00						1.00		1.00	
<0 - 0.9	1.20	1.05 - 1.36					1.20	1.06 - 1.37	1.17	1.02 - 1.34
1 - 1.9	1.45	1.25 - 1.69					1.43	1.24 - 1.66	1.38	1.18 - 1.61
2 - 2.9	1.62	1.39 - 1.89					1.60	1.38 - 1.86	1.55	1.33 - 1.81
3 people or more	1.88	1.61 - 2.21					1.84	1.56 - 2.16	1.76	1.49 - 2.09
Number of dentists (full time equivalent)										
1 person	1.00		1.00		1.00		1.00		1.00	
<1-1.9	1.14	1.05 - 1.23	1.11	1.04 - 1.20	1.10	1.02 - 1.18	1.04	0.97 - 1.11	1.03	0.96 - 1.11
2 people or more	1.21	1.09 - 1.34	1.15	1.04 - 1.26	1.14	1.03 - 1.26	0.99	0.89 - 1.09	0.95	0.87 - 1.05
Sex										
Male	1.00		1.00		1.00		1.00		1.00	
Female	1.18	1.13 - 1.24	1.17	1.13 - 1.22	1.17	1.13 - 1.22	1.17	1.13 - 1.22	1.17	1.13 - 1.22
Age (years)										
20-29	1.00		1.00		1.00		1.00		1.00	
30-39	1.00	0.92 - 1.10	1.01	0.93 - 1.09	1.01	0.93 - 1.10	1.00	0.92 - 1.09	1.00	0.92 - 1.09
40-49	0.98	0.91 - 1.05	1.00	0.93 - 1.07	1.00	0.93 - 1.07	0.99	0.92 - 1.07	1.00	0.93 - 1.07
50-59	0.96	0.87 - 1.04	0.98	0.90 - 1.07	0.98	0.90 - 1.07	0.99	0.90 - 1.08	0.98	0.90 - 1.08
60-69	1.01	0.92 - 1.11	1.06	0.97 - 1.15	1.06	0.98 - 1.15	1.07	0.98 - 1.17	1.07	0.98 - 1.16
70-79	0.97	0.87 - 1.07	1.05	0.96 - 1.15	1.05	0.96 - 1.15	1.07	0.97 - 1.17	1.07	0.97 - 1.17
80-89	0.91	0.81 - 1.01	1.03	0.93 - 1.14	1.03	0.93 - 1.14	1.06	0.96 - 1.17	1.06	0.96 - 1.17
90+	0.63	0.35 - 1.13	0.84	0.48 - 1.45	0.84	0.49 - 1.45	0.85	0.50 - 1.46	0.86	0.50 - 1.47
Subjective economic status										
Low	1.00		1.00		1.00		1.00		1.00	
Middle	1.13	1.07 - 1.19	1.09	1.04 - 1.14	1.09	1.04 - 1.14	1.08	1.03 - 1.13	1.08	1.03 - 1.13
High	1.23	1.14 - 1.32	1.15	1.08 - 1.24	1.15	1.07 - 1.23	1.15	1.07 - 1.23	1.14	1.07 - 1.22
Years of education										
≦9	1.00		1.00		1.00		1.00		1.00	
10-12	1.19	1.09 - 1.29	1.13	1.04 - 1.23	1.13	1.04 - 1.23	1.13	1.05 - 1.23	1.14	1.05 - 1.23
14-15	1.31	1.20 - 1.43	1.22	1.13 - 1.32	1.22	1.13 - 1.32	1.22	1.13 - 1.32	1.22	1.13 - 1.32
≧16	1.32	1.21 - 1.44	1.27	1.17 - 1.38	1.27	1.17 - 1.38	1.27	1.17 - 1.38	1.28	1.18 - 1.39
Random effects (SE)										
Prefecture	0.02**	(0.01)	0.01	(0.01)	0.01	(0.00)	0.02	(0.00)	0.02	(0.00)
Dental clinic	0.12**	(0.02)	0.10	(0.01)	0.09	(0.01)	0.07	(0.01)	0.06	(0.01)
Median prevalence ratio (MPR)	MPR	95%CI	MPR	95%CI	MPR	95%CI	MPR	95%CI	MPR	95%CI
Prefecture	1.14**	1.10 - 1.18	1.10	1.10 - 1.18	1.10	1.10 - 1.18	1.14	1.10 - 1.18	1.14	1.10 - 1.18
Dental clinic	1.39**	1.33 - 1.45	1.35	1.29 - 1.41	1.33	1.29 - 1.39	1.29	1.24 - 1.33	1.26	1.24 - 1.33
Variance partition coefficient(VPC)	VPC		VPC		VPC		VPC		VPC	
Prefecture	0.6%**		0.3%		0.3%		0.6%		0.6%	
Dental clinic	3.5%**		2.9%		2.7%		2.1%		1.8%	

* The reason of dental visit were dichotomized as follows: (0) treatment and (1) regular check-ups, with or without treatment.

**Random effects, MPR, and VPC for the null model; the model includes only a constant.
